# Integrating artificial intelligence into the modernization of traditional Chinese medicine industry: a review

**DOI:** 10.3389/fphar.2024.1181183

**Published:** 2024-02-23

**Authors:** E. Zhou, Qin Shen, Yang Hou

**Affiliations:** ^1^ Yuhu District Healthcare Security Administration, Xiangtan, China; ^2^ Department of Respiratory Medicine, Hunan Provincial People’s Hospital (The First Affiliated Hospital of Hunan Normal University), Changsha, China; ^3^ Xiangya School of Pharmaceutical Sciences, Central South University, Changsha, China

**Keywords:** traditional Chinese medicine, artificial intelligence, drug discovery, data mining, quality standardization, industry technology

## Abstract

Traditional Chinese medicine (TCM) is the practical experience and summary of the Chinese nation for thousands of years. It shows great potential in treating various chronic diseases, complex diseases and major infectious diseases, and has gradually attracted the attention of people all over the world. However, due to the complexity of prescription and action mechanism of TCM, the development of TCM industry is still in a relatively conservative stage. With the rise of artificial intelligence technology in various fields, many scholars began to apply artificial intelligence technology to traditional Chinese medicine industry and made remarkable progress. This paper comprehensively summarizes the important role of artificial intelligence in the development of traditional Chinese medicine industry from various aspects, including new drug discovery, data mining, quality standardization and industry technology of traditional Chinese medicine. The limitations of artificial intelligence in these applications are also emphasized, including the lack of pharmacological research, database quality problems and the challenges brought by human-computer interaction. Nevertheless, the development of artificial intelligence has brought new opportunities and innovations to the modernization of traditional Chinese medicine. Integrating artificial intelligence technology into the comprehensive application of Chinese medicine industry is expected to overcome the major problems faced by traditional Chinese medicine industry and further promote the modernization of the whole traditional Chinese medicine industry.

## 1 Introduction

Traditional Chinese medicine (TCM) is an ethnopharmacology system composed of thousands of years’ practical experience of the Chinese nation and TCM prescriptions. It has rich and effective natural resource and accumulated many valuable experiences in fighting diseases (Ma et al., 2021; Deng et al., 2023). Modern research has confirmed that TCM play an important role in the medical field, as evidenced by its role in enhancing anti-tumor effect and treating various disease (Augustin et al., 2020; Bai et al., 2022). A large number of clinical studies have consistently demonstrated that some components in TCM show remarkable curative and relieving effect, especially for chronic and complex diseases that cannot be solved by traditional medical solutions (Huang and Xuan, 2020; Leung and Xu, 2020; Cheng et al., 2022; Chen et al., 2023; Huang et al., 2023). Due to the potential side effects associated with many modern chemically synthesized drugs, there is a growing need to seek safer alternative medications. Exploring natural plant sources has become a significant way in drug discovery. TCM has attracted more and more attention from medical and healthcare workers due to its multi-component, multi-target and multi-channel pharmacological effects (Shi et al., 2022). Especially in the relentless struggle against the COVID-19 epidemic, the important role of TCM has been firmly confirmed. For example, formulas such as Lianhua Qingwen capsules (Forsythiae Fructus [Oleaceae; Forsythia suspensa (Thunb.) Vahl], Lonicerae Japonicae Flos [Caprifoliaceae; *Lonicera japonica* Thunb.], Ephedrae Herba [Ephedraceae; Ephedra equisetina Bunge], Armeniacae Semen Amarum [Rosaceae; Prunus armeniaca L.], Gypsum Fibrosum, Isatidis Radix [Brassicaceae; Isatis tinctoria L.], Dryopteridis Crassirhizomatis [Polypodiaceae, Dryopteris crassirhizoma Nakai], Houttuynia cordata [ Saururaceae, Houttuynia cordata Thunb.], Pogostemonis Herba [Lamiaceae, Pogostemon cablin (Blanco) Benth.], Rhei Radix Et Rhizoma [Polygonaceae, Rheum offcinale Baill.], Rhodiolae Crenulatae [Crassulaceae, Rhodiolae Crenulatae Radixet Rhizoma], Menthae Haplocalycis Herba [Lamiaceae, Mentha canadensis L.], Glycyrrhizae [Fabaceae, Glycyrrhiza glabra L.]) (Liu et al., 2021) and Jinhua Qinggan granules (Lonicerae Japonicae Flos [Caprifoliaceae; *L. japonica* Thunb.], Gypsum Fibrosum, Ephedrae Herba [Ephedraceae; Ephedra equisetina Bunge], Armeniacae Semen Amarum [Rosaceae; Prunus armeniaca L.], Scutellariae Radix [Lamiaceae, Scutellaria baicalensis Georgi], Forsythiae Fructus [Oleaceae, Forsythia suspensa (Thunb.) Vahl], Thunberg Fritillary [Liliaceae, Fritillaria thunbergia Miq.], Anemarrhena [Liliaceae, Anemarrhena asphodeloides Bunge], Arctium lappa [Asteraceae, Arctium lappaL.], Artemisiae Annuae Herba [Asteraceae, Artemisia annua L.], Menthae Haplocalycis Herba [Lamiaceae, Mentha canadensis L.], Glycyrrhizae [Fabaceae, Glycyrrhiza glabra L.]) (Gajewski et al., 2021) have deepened people’s understanding of the importance of TCM. However, despite the extensive pharmacological activities exhibited by TCM, there remains a lack of a modern scientific basis for reaching mechanism behind its active components and the compatibility principles (Liu and Guo, 2020). Furthermore, the standard quality system of TCM has not yet been established. These are still the primary issues and challenges in the research of TCM.

Artificial intelligence, referred to as AI for short, is a rapidly developing new technical science. Its theories, methods and technical applications simulate, extend and expand human intelligence (Abolaban, 2023). AI uses various algorithms for machine learning and deep learning, including support vector machine (SVM), Bayesian network, artificial neural networks (ANNs), random forest (RF), convolutional neural networks (CNNs) and more (Santos et al., 2021; Yang et al., 2022). Over recent decades, AI has made significant breakthroughs in various fields, such as finance (Ahmed et al., 2022), traffic (Shaygan et al., 2022), electrical automation (Sami, 2022), medical care (Yahyaoui et al., 2023), biomedical research and others (Diakiw et al., 2022). Notably, the application of AI in TCM industry has become increasingly prevalent in recent years. Traditional drug development usually starts with the targets and mechanisms verified by preclinical research, which is time-consuming and expensive (Wouters et al., 2020). AI technology has significantly improved the reliability and accuracy of diagnosis, target screening and new drug research, which has greatly aroused the extensive concern and interest of scholars (Liu et al., 2021; Chen et al., 2024). Many studies show that artificial intelligence plays an important role in the quality evaluation of TCM, drug target discovery, optimization of compatibility and medical diagnosis of TCM, and AI pharmacy is becoming a very interesting research topic in academic circles (Xu et al., 2021; Gong et al., 2021; Li et al., 2022; Lin et al., 2022).

**FIGURE 1:**
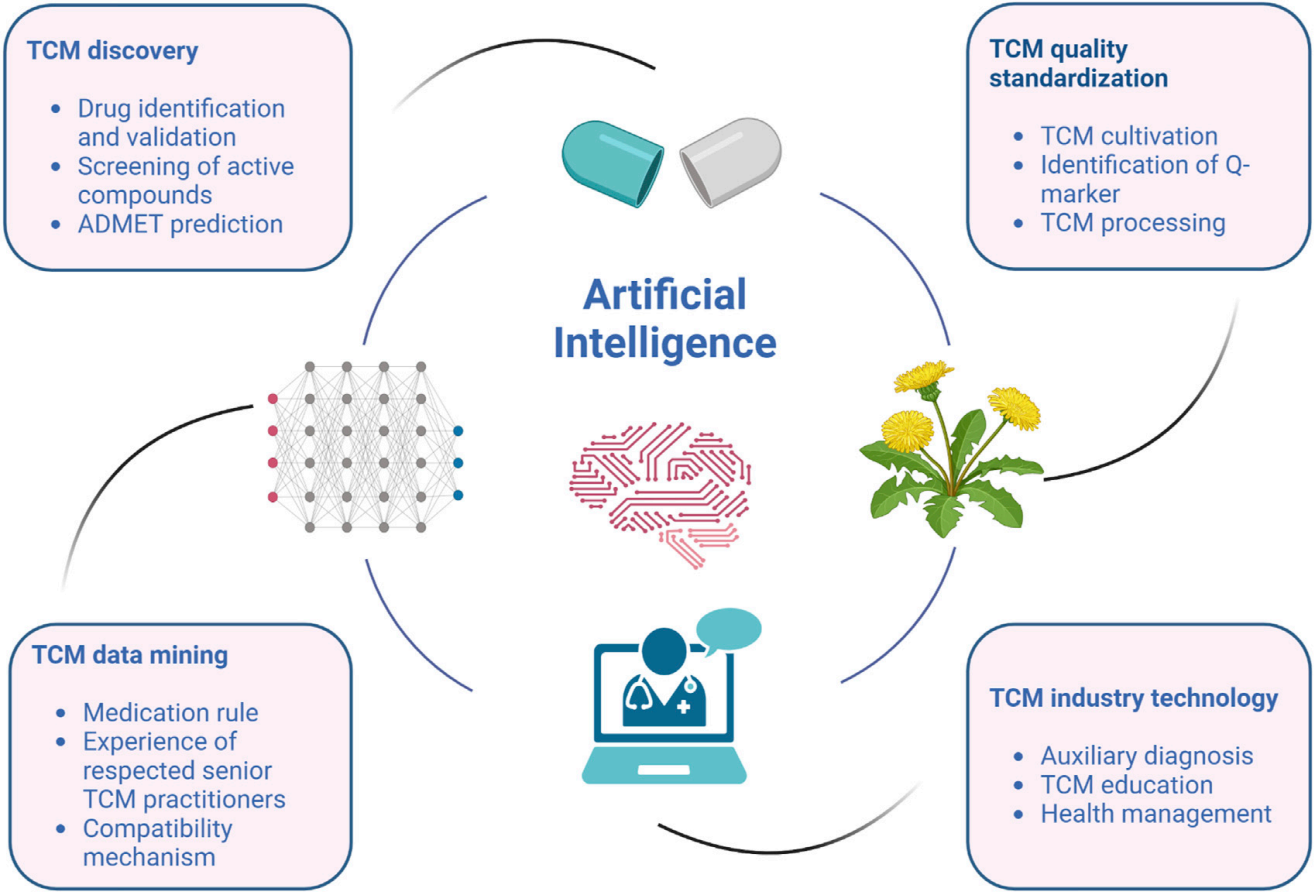
Application of AI in various aspects of TCM industry.

With the continuous progress of computer technology, the application of AI in the modernization of TCM has covered many aspects, including new drug discovery, data mining, quality standardization and some industry technologies, as shown in Figure 1. For example, Lam et al. explored the application mode of TCM in children’s cancer care by using association rule mining technology (Lam et al., 2022a). Network analysis has been used to identify the toxic components and quality marks of TCM, as well as to reveal the effective components and mechanism of action of TCM prescriptions (Ma et al., 2021). Wang and others proposed that AI can help doctors make more accurate clinical decisions and provide assistance in basic TCM medical care (Wang et al., 2022a). The application of AI in these different links is interrelated and jointly promotes the development and innovation of TCM. However, most literature focus on introducing the application of AI in a single link of TCM development. Therefore, this paper aims to comprehensively summarize the application of AI in various aspects of the modernization process of TCM industry, reveal the potential of AI technology and provide a comprehensive perspective for understanding the value of AI in the field of TCM.

## 2 Application of AI in TCM discovery

### 2.1 The process of drug discovery

**FIGURE 2:**
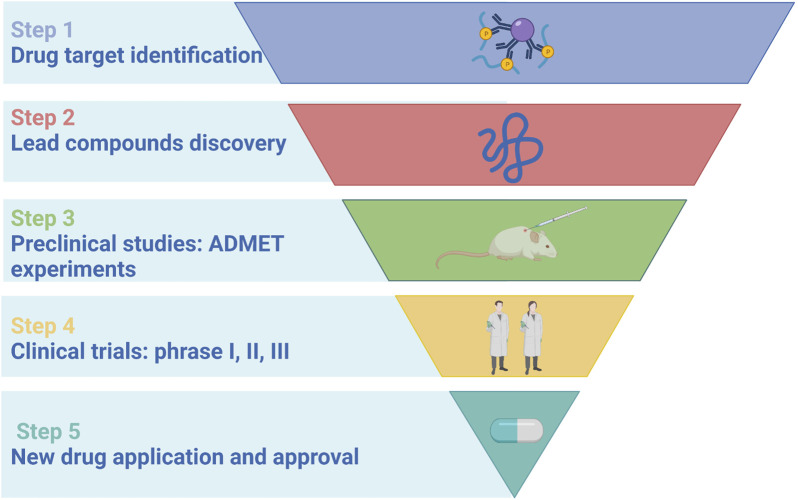
Major steps of drug discovery process.

The process of traditional drug discovery includes drug targets identification, lead compounds discovery, preclinical studies (ADMET prediction), clinical trials (phase I, II, III) and new drugs application and approval (Miller et al., 2023), as shown in Figure 2. The process of new TCM discovery is similar, which needs preclinical research and clinical research before it can be approved. However, due to the unique properties of botanical drugs different from chemical drugs, FDA issued a set of regulatory guidelines different from non-botanical drugs, namely, Botanical Fruit Development Guidance for Industry guidance. This guidance proposed that based on a large number of people’s medication experience and history, the requirements for pharmaceutical and toxicological research in the initial clinical trial in IND stage were relatively relaxed, but as a drug listed (NDA application), the safety and effectiveness must meet the same technical guidelines.

The composition and biological action network of TCM are extremely complex, and the number of prescriptions is huge (Guo et al., 2020). Generally, the research and development of new TCM drugs is carried out through clinical empirical prescriptions and long-term human experience, which is inefficient, consumes resources and has unclear action mechanism. These constraints urge researchers to find new ways of TCM discovery. The emergence of big data and AI technology has brought new ideas and new directions for TCM discovery. Using AI technologies such as machine learning, deep learning and natural language processing, TCM literature, clinical cases and TCM databases can be mined, which can significantly improve the processing efficiency of pharmaceutical data and information, find potential TCM components and mechanisms, and greatly improve the efficiency and accuracy of TCM discovery (Zhou and Wang, 2014; Xu et al., 2019).

### 2.2 Drug target identification and validation

In view of its efficient and fast data analysis ability, the application of big data in TCM discovery is mainly aimed at targets identification of TCM. Machine learning based on AI can identify and verify the target protein related to disease pathology by mining a series of biomedical databases, which plays a vital role in the initial stage of new drug discovery. Liu et al. built the multi-target-based polypharmacology prediction model (mTPP) by using Multi-layer Perceptron (MLP), Support Vactor Regression (SVR), Decision Tree Regressor (DTR), and Gradient Boost Regression (GBR) algorithms to predict 20 candidates with potential effects against drug-induced liver injury (DILI) (Liu K. et al., 2022). The accuracy of the model was verified by 2 of the 20 candidates, which can be used to explore the relationship between multi-target effect and overall drug efficacy. He et al. proposed a QSAR model composed of several ML algorithms to study the potential candidates of TCM that can be well docked with protein targeting Alzheimer’s disease (He et al., 2020). Furthermore, Pun et al. utilized PandaOmics, an AI-driven target discovery platform, to analyze the expression profiles of central nervous system (CNS) samples from public datasets, and direct iPSC-derived motor neurons (diMNs) from Answer ALS, and identified 28 potential therapeutic targets for ALS and verified 8 unreported genes (Pun et al., 2022). These AI-driven methodologies speed up the target discovery process and significantly narrow down the screening range of candidate drugs for downstream experiments.

### 2.3 Screening of active compounds

After identifying the drug target, the next step is to search for suitable drug molecules that can effectively interact with the target (Jiménez-Luna et al., 2021). Firstly, hit compounds with preliminary activity for specific targets were screened from a large number of drug molecules, subsequently one or several compounds with better activity were determined as lead compounds. These compounds can be derived from different origins, including natural substances found in animals, plants and marine life, or even known compounds (Atanasov et al., 2021). AI is helpful to guide lead compounds discovery with pharmacological activities from natural sources such as plants and microorganisms (Li et al., 2022; Palermo, 2023). Moreover, AI can identify the complex components of TCM, and explore active components in TCM more accurately and quickly. Xu et al. screened 74 active substances and 2128 herbal prescriptions for adjuvant treatment of gastric cancer from TCM public databases by using association rule mining (ARM). They studied the key substances in the source prescriptions through network pharmacology and further verified that the prescriptions can inhibit tumor progression through *in vitro* and *in vivo* signal pathways, which indicated the accuracy of machine learning (Xu et al., 2021). In addition, It is capable to effectively search for known active compounds by simulating the interaction between target and candidate drug molecules through virtual screening (Gorgulla et al., 2022). Tang et al. established a deep learning workflow -Deep Docking to screen large molecular libraries, and successfully screened two active compounds as new adenosine receptor antagonists in a deep learning model based on docking of about 3 million compounds, which was proved to be effective in vitro experiments (Tang et al., 2023). Lin et al. identified three new renal glutaminase inhibitors through virtual screening based on structure, and further confirmed the inhibitory effects of these three compounds through enzyme and cell viability determination experiments (Lin et al., 2022). These studies have revealed the important role and great potential of AI in exploring the composition of TCM and predicting the active ingredients.

### 2.4 ADMET prediction

Following the identification of lead compounds, it is necessary to repeatedly test their ADMET characteristics in genetic toxicity model and animal behavior model. This is one of the most important steps of the entire drug discovery processes (Ajala et al., 2023). Adverse pharmacokinetics and toxicity characteristics of candidate drugs are the common reasons for the failure of drug discovery. By carefully selecting and optimizing lead compounds according to their ADMET properties in the early stage of drug discovery, it is possible to greatly improve the success rate and reduce the later cost. DAINA et al. demonstrated the SwissADME network tool, which is an efficient and free resource for predicting physicochemical properties, pharmacokinetics, drug similarity and drug chemical compatibility (Daina et al., 2017). Bakchi et al. also made a valuable summary on the practical application of SwissADME web tool in drug design and development (Bakchi et al., 2022). Additionally, Handi et al. evaluated the drug-like potential of all designed compounds by calculating ADMET properties, and obtained suggestions for designing new drug candidates (Hadni and Elhallaouia, 2022). These studies show the importance of AI technology in ADMET prediction. In the follow-up clinical research, AI technology narrows the experimental scope, reduces the probability of clinical failure and significantly decreases the cost of new drug discovery (Moingeon et al., 2022).

### 2.5 Limitations and challenges of AI in TCM discovery

AI has great potential to accelerate new TCM discovery by optimizing the entire drug discovery process. A summary of related research findings is provided in Table 1. By establishing connections between TCM and diseases, AI can quickly screen out compounds with high activity as TCM regulating agents to regulate various diseases in human system. Despite the widespread popularity of AI-driven TCM discovery research, there are still some limitations and deficiencies need to be acknowledged. For instance, while AI aids in the computer-aided design or discovery of TCM molecules, it is necessary to verify their effectiveness in the target diseases through clinical experiments. In some studies, only the preliminary prediction results of computer analysis are often given, but there is a lack of experimental verification of the screened candidate drugs. Therefore, the importance of subsequent clinical trials cannot be overstated.

**TABLE 1:** Application of AI in TCM discovery.

Aim of study	AI methods	Results	REF
Screen and validate the potential inhibitors of diabetes mellitus from TCM Database	Machine learning regression models, and deep neural network models	Illustrated that Hypecoum leptocarpum [Papaveraceae; Hypecoum leptocarpum Hook.f. & Thomson] might be a potential and effective medicine herb for the treatment of DM	Gong et al. (2021)
Identify potential inhibitor against phospholipase A2 (PLA2)	Virtual screening and molecular docking	Confirmed that Scutellarin [Lamiaceae; Scutellaria baicalensis Georgi] (TCM3290) had a potent snake venom neutralizing capacity	Chinnasamy et al. (2020)
Discover the molecular mechanism of Qu’s formula in treating endometriosis and to explore the potential drug targets	Network pharmacology, molecular docking	Confirm the clinical effectiveness of QUF	Wu et al. (2022)
Discover multi-target compounds with anti-inflammation activity from TCM	TCM target-effect relationship spectrum, virtual screening model	Seven compounds were chosen with multiple targeted activity based on the TCM-TERS	Huo et al. (2022)
Explore effective TCM against porcine reproductive and respiratory syndrome (PRRS)	Computer-aided drug design technology, virtual screening	Suggested that Andrographolide [Acanthaceae; Andrographis paniculata (Burm.f.) Nees] and Potassium Dehydrographolide Succinate are promising PRRSV inhibitors *in vitro*	Su et al. (2021)
Explore an integrated new approach for finding lead compounds that inhibit galectin-3	Deep learning-based algorithm	Found that the active ingredients 1,2-Dimethylbenzene and Typhic acid contained in Crataegus pinnatifida Bunge [Rosaceae; Crataegus Fructus] and Typha angustata [ Typhaceae; Typha domingensis Pers.] might be the effective inhibitors of neurodegenerative diseases	Deng et al. (2020)
Explore new targets and new active components of TCM in kidney stones	Molecular docking	Four active ingredients were predicted to have effects on treating kidney stones	Zhang et al. (2023)
Screen a drug targeting thioredoxin reductase (TrxR) from TCM database	Molecular docking	Identified two compounds as structure-based lead inhibitors against TrxR	Lata and Akif (2021)
Explore the possible target pathways for Gentianine [Gentianaceae; Gentiana scabra Bunge] in anti-ischemic stroke	Machine learning, molecular docking	Gentianine [Gentianaceae; Gentiana scabra Bunge] could be used as a potential anti-ischemic stroke agent by suppressing inflammatory responses *via* TLR4/NF-κB signaling pathway	Wang et al. (2021a)

TCM usually lacks a deep understanding of the molecular mechanism of drugs and adopts holistic treatment methods, which brings challenges to the application of AI (Guo et al., 2020; Mu et al., 2023). In addition, there are some problems in the database of TCM, such as different quality and poor management, which may cause false positive results in computer network analysis (Wang et al., 2022; Li et al., 2023). The lack of high-quality and comprehensive database of Chinese herbal medicines and formulas hinders the training and verification of AI models. In order to overcome these limitations, it is necessary to strengthen the quality control of TCM database and establish a unified data standard and classification system of TCM (Wang et al., 2023).

## 3 Application of AI in data mining of TCM

### 3.1 TCM medication rule

TCM industry has accumulated a vast amount of prescription data through years of medical practice, typically composed of multiple drugs and their respective dosages. These TCM prescriptions are generally created by experienced TCM practitioners, who possess a wealth of medical wisdom. However, due to the limited number and time of these famous doctors, they cannot meet the growing needs of more patients. Therefore, people began to look for more efficient ways to assist TCM doctors in prescription optimization (Xian et al., 2023). Data mining technology, an interdisciplinary branch of computer science involving the cross use of AI, machine learning, statistics and database, play a vital role in analyzing large-scale TCM prescription datasets and identify potential patterns among various drugs (Birjandi and Khasteh, 2021). Data mining technology can discover the hidden rules between things from massive data, and is regarded as one of the most effective data analysis methods at present. In data mining technology, some commonly methods used include frequency analysis, correlation analysis, complex network analysis and cluster analysis (Chen et al., 2023; Rastogi and Bansal, 2023). By using these data mining technologies to analyze clinical data and literature of TCM, AI algorithms can identify the patterns of herbs used, the combinations methods and the dosage patterns of herbs (Sun et al., 2020a; Lam et al., 2022a). This analytical approach is invaluable in determining the most effective herbal combinations for treating specific diseases and the ideal dosage of each constituent of herbs (Sun et al., 2020a). Furthermore, AI can also be used to identify potential adverse reactions between different herbs, thus ensuring the safety of TCM prescriptions (Qiao et al., 2022). TCM data mining effectively summarize and inherit the rich experience of TCM by finding frequent patterns and association rules among medicinal components. However, it is worth noting that these observed patterns often lack a comprehensive pharmacological explanation behind them and need further research to uncover the underlying mechanism.

### 3.2 Experience of respected senior TCM practitioners

The theoretical basis of TCM represents rich and invaluable treasure house of medical experience that has been passed down for thousands of years. Exploring TCM theory and ancient literature deeply is crucial for promoting the inheritance and development of this medical cultural heritage (Xu et al., 2020; Chaoping et al., 2022). However, numerous ancient TCM documents have been destroyed and scattered across the country, with their compilation methods differing from modern standards, making them difficult to access and query. Data mining is the intersection of AI and mathematical statistics, and it is a new discipline that is expected to solve these challenges. Using machine learning technology of AI, it is feasible to digitize ancient Chinese herbal books and the experiences of famous TCM experts, and dig relevant information for in-depth analysis, and provide valuable insights for clinical diagnosis and treatment (Liao et al., 2020). It is worth noting that the introduction of innovative model greatly improves the accuracy of TCM prescription recommendation. Zhao et al. proposed the multi-graph convolution network (MGCN) prescription recommendation model, while Dong et al. proposed the TCM prescription recommendation model (TCMPR), both of which showed impressive performance (Dong et al., 2022; Zhao et al., 2022). In order to further explore the synergy between AI and TCM, Zhi et al. built TCMBERT model, which is a special intelligent model for training TCM books to generate TCM prescriptions (Liu et al., 2022b). Deng and others used data mining to verify and popularize the clinical experience and prescription of Xiong Jibo, a master of TCM, in treating arthralgia syndrome (Wenxiang et al., 2022). In addition, Zhou et al. put forward FordNet, which is an intelligent TCM recommendation system integrating phenotypic and molecular data. The system analyzed more than 20,000 electronic health records managed by Li Jiren, a TCM expert, and achieved an excellent learning effect (Zhou et al., 2021).

Although AI algorithm provides extraordinary potential, it is still a complex task to completely capture and simulate the subtle professional knowledge of TCM, involving complex individual differences and comprehensive judgments. Therefore, when AI is applied to the field of experience mining, it is still essential to integrate professional clinical experience and the judgments of respected senior TCM practitioners.

### 3.3 TCM compatibility mechanism

Chinese herbal compound prescription plays a synergistic or antagonistic role through compatibility, making it an integral part of Chinese herbal medicine theory (Xie et al., 2021). TCM formula based on compatibility theory provides a drug therapy method to solve diseases through the interaction of various compounds, which reflects the concept of combined therapy in modern medicine (Wang et al., 2022c). The human body is a complex biological system, and it often suffer from diseases caused by multiple factors. TCM has shown remarkable curative effect on complex diseases through multiple targets, multiple compounds and multiple ways (Qiao et al., 2021; Huang et al., 2023). In the research of modernization of TCM, many scholars have accumulated a large amount of pharmacological and action mechanism data of TCM and its prescriptions through AI technology, which provide valuable information resources for the clinical application of TCM prescriptions (Yang et al., 2020; Xiong et al., 2022; Xue et al., 2022). For example, Ding et al. reveals the mechanism of activity of Ge-Gen-Qin-Lian decoction [Pueraria montana var. lobata [Fabaceae, Pueraria montana var. lobata (Willd.)], Scutellariae Radix [Lamiaceae, Scutellaria baicalensis Georgi], Glycyrrhizae [Fabaceae, Glycyrrhiza glabra L.], Coptis chinensis [Ranunculaceae, Coptis chinensis Franch.]] against LPS-induced acute lung injury based on system biology method (Ding et al., 2020). A network pharmacological framework was proposed by Wang et al. to quantify the interaction between herbal pairs (Wang et al., 2021). These studies are of great significance to explore the molecular mechanism of TCM prescription for complex diseases by using AI technology. See Table 2 for more related research.

**TABLE 2:** Application of AI in TCM data mining.

Aim of study	AI methods	Results	REF
Propose a multi-graph convolution network (MGCN) prescription recommendation model	Multi-graph convolutional network	MGCN significantly improved the accuracy of TCM herbal prescription recommendations	Zhao et al. (2022)
Propose a TCM prescription recommendation model (TCMPR)	Deep learning	TCMPR has high performance on TCM prescription recommendation	Dong et al. (2022)
Generate TCM prescriptions from a few medical records and TCM documentary resources	a two-stage transfer learning model, TCMBERT model	TCMBERT model outperforms the state-of-the-art methods in all comparison baselines on the TCM prescription generation task	Liu et al. (2022b)
Examine and propagate the medication experience and group formula of TCM Master XIONG Jibo in diagnosing and treating arthralgia syndrome (AS)	Frequency analysis, association rule analysis, cluster analysis, and visual analysis	Customized NLP model could improve the efficiency of data mining in TCM	Wenxiang et al. (2022)
Propose an intelligent formula recommendation system (FordNet)	Deep learning, convolution neural network	FordNet can learn from the effective experience of TCM masters and get excellent recommendation results	Zhou et al. (2021)
Propose mechanism about a TCM prescription	PageRank algorithm, network pharmacology	Provided a new unsupervised learning strategy for polypharmacology research about TCM	Xiong et al. (2022)
Explore the mechanism of eight classic TCM formulae in the treatment of different types of coronary heart disease	Screening, network clustering, hierarchical clustering, network topology	Showed that each formula’s targets were significantly correlated with CHD associated genes and overlapped with the targets of 9 classes of drugs for cardio vascular diseases (CVD) to some degree	Yang et al. (2020)
Explore the effects and mechanisms of Ge-Gen-Qin-Lian decoction treatment in acute lung injury	Network pharmacology	Suggested that GQD did have a better therapeutic effect on acute lung injury	Ding et al. (2020)
Quantify the interactions in herb pairs	Network-based modeling	Provided a network pharmacology framework to quantify the degree of herb interactions	Wang et al. (2021b)
Explore the patterns of TCM use and its efficacy in children with cancer	Association rule mining	ARM showed that Radix Astragali, the most commonly used medicinal herb (58.0%), was associated with treating myelosuppression, gastrointestinal complications, and infection	Lam et al. (2022b)
Explore the potential therapeutic effect of TCM on coronavirus disease 2019 (COVID-19)	Data mining, frequency and association analysis, network pharmacology analysis, bioinformatics analysis	Collected a total of 173 prescriptions which were involved in the anti-inflammatory, anti-viral, and neuroprotective effects	Sun et al. (2020b)
Investigate the potential mechanism of Biyuan Tongqiao granule (BYTQ) against allergic rhinitis (AR)	Network pharmacology	Found the potential protein targets and mechanism for BYTQ to treat AR	Wang et al. (2024)

AI plays an indispensable role in advancing the field of TCM by efficiently excavating and promoting the sustainable utilization of TCM resources. However, it is important to admit that each data mining method has its inherent limitations, and the mining process requires human-computer interaction. Under the background of professional knowledge of TCM, appropriate mining methods are selected for different mining objects. Facing the complexity of TCM data, traditional statistical analysis tools and simple data mining technology can no longer meet the needs of TCM information development. Therefore, deeper AI integration is necessary for further analysis and processing of these massive data.

## 4 Application of AI in quality standardization of AI

### 4.1 TCM cultivation

The quality of TCM available in the market is often inconsistent, with various issues such as adulteration, mixed use, heavy metal pollution, and artificial coloring (Chen et al., 2021). These problems not only affect the efficacy and safety of TCM, but also seriously hinder the high-quality development of TCM industry. The quality challenge of TCM has become a core problem to be solved urgently (Tu et al., 2021). High-quality raw materials are the basis to ensure the quality of TCM products. However, TCM growers lack understanding of the application of TCM and scientific cultivation techniques, and insufficient seed screening and control measures hinder the efforts to improve TCM quality.

Through the application of intelligent agricultural technology, the best planting technology of TCM can be screened out by using the previous planting data of TCM, which helps growers to produce higher quality TCM products (Wakchaure et al., 2023). Machine learning is a promising technology, which can automatically extract the key insight from a large number of data sets related to the origin of TCM, including the basic climatic conditions for cultivating a variety of TCM, such as temperature, humidity and light requirements (Wakchaure et al., 2023). In addition, AI technology can identify different kinds of TCM through pattern recognition, which provides an effective strategy for the identification of different TCM and greatly shortens the identification time (Tan et al., 2020; Yue et al., 2021; Liu C. et al., 2022). AI technology has also promoted the establishment of a comprehensive database, collecting information about the types, origins, efficacy, chemical components, pharmacological effects, cultivation techniques and processing methods of TCM. Such a database is convenient for the management, traceability and resource investigation of TCM (Wongchai et al., 2022). Big data analysis driven by AI is based on medicinal materials samples and covers many aspects, including authenticity identification, origin tracking and quality prediction (Guo et al., 2022). The application of AI technology in TCM varieties, origin distribution, and cultivation techniques has achieved remarkable results, which provides strong technical support for the standardization of TCM cultivation.

### 4.2 Identification of quality marker (Q-marker)

Quality is an important guarantee for the clinical efficacy and safety of TCM. However, the standardization evaluation of TCM quality is still an unsolved challenge, making quality control a key issue in the field of TCM (Wang et al., 2022). The quality inspection of TCM usually includes appearance and composition. Appearance detection involves the evaluation of the color, shape, and smell of Chinese herbal medicines. In contrast, component detection is more critical as it can determine the components of TCM and detects heavy metals and pesticide residues. In view of the fact that TCM is usually composed of various components, and each component has different pharmacological effects, many methods have been adopted to monitor the quality of TCM. These methods include chromatography, spectroscopy, fingerprint technology and mass spectrometry imaging technology (Cheng et al., 2021; Shen et al., 2021; Shi et al., 2021; Jiang et al., 2022). However, these research methods have some limitation in solving the complex components and efficacy of TCM, and often lack objectivity in evaluating the effectiveness and safety of TCM (Zhang et al., 2022). Therefore, there is an urgent need for more accurate and effective detection techniques and methods to promote the innovation of TCM quality, as well as to establish a scientific and feasible modern quality control standard.

In order to standardize the quality research of TCM, academician Liu Changxiao established a new quality research model of TCM-Q marker according to the biological characteristics, manufacturing technology and compatibility theory of TCM system (Liu et al., 2017). The Q-marker of TCM is a marked chemical substances inherent in TCM or formed in the processing stage, which is closely related to the functional attributes of TCM and can reflect the safety and effectiveness of TCM (Zhang et al., 2022). The Q-markers of TCM provides a new prospective and direction for quality control of TCM, which is helpful to establish a comprehensive quality control and traceability system for the production of TCM species (Dong et al., 2023). However, the discovery of these markers is a challenging process and requires a lot of basic research work. The combination of AI technology and Q-marker shows great application potential in quality control of TCM. Zhao et al. established a HPLC fingerprinting method centered on predicting Q-marker of Atractylodis Rhizoma [Asteraceae; Atractylodis macrocephalae Rhizoma] through network pharmacology (Zhao et al., 2023). Cheng et al. screened out the potential anti-AS mechanisms and chemical Q-markers based on the combination of serum pharmacochemistry and network pharmacology (Cheng et al., 2022). He et al. put forward a new strategy of multi-dimensional “radar chart” mode discover the Q-markers of Qiliqiangxin Capsule (QLQX), which provided a new idea for TCM quality management (He et al., 2021).

### 4.3 TCM processing

The quality of TCM mainly depends on two key factors, which are raw materials and processed drugs. Raw materials are the basis of quality, while the specific curative effect is determined by processing method (Tsai et al., 2023). The processing of TCM is a pharmaceutical technology based on the theory of TCM, syndrome differentiation, the inherent properties of drugs and the unique requirements for compounding and preparation. The purpose is to retain therapeutic ingredients while eliminating ineffective or potentially harmful ingredients (Zhou et al., 2022). TCM is mainly administered in the form of slices or pieces. Chinese herbal pieces refer to TCM that can be directly used in clinical prescription after being processed according to TCM theory. AI technology, especially deep learning and neural intelligent network, can automatically train and intelligently identify Chinese herbal pieces. This is helpful for fine management in the whole processing process cycle of Chinese herbal pieces, ensuring its standardization and quality stability (Xu et al., 2021).

TCM is usually processed into decoction to release its therapeutic efficacy (Wang et al., 2023). The decocting process is a crucial step that affects the quality and curative effect of TCM. Through different decocting methods, its efficacy and therapeutic effect can be maximized (Peng et al., 2022). Decocting medicine is a delicate and complicated process, and it is challenging for the general public to accurately master it due to their limited understanding of TCM. Many researchers have begun to use AI and machine-based decocting system that can be customized on a large scale. Through AI and other modern technologies, the intelligent decocting center can be integrated with the computer-controlled decocting equipment system, enabling the visualization and online monitoring of the decocting process of TCM. This standardized the production of TCM decoction and enhanced the quality and curative effect of TCM.

Quality control plays an important role in ensuring the safe and effective use of TCM. AI has great potential in improving the cultivation practices of TCM, establishing Q-markers as a standardized model for quality assessment of TCM and simplifying the processing of TCM. Other research results are presented in Table 3. The quality control of TCM involves several aspects, including planting, processing and component analysis. Lack of unified standards and norms may lead to differences between AI models and methods developed by different research teams, which hider the comparison and sharing of results. Although AI can effectively manage a large number of data and extraction patterns, it still needs to be combined with expert knowledge and experience in the fields of TCM to promote the high-quality development of TCM.

**TABLE 3:** Application of AI in quality standardization of TCM.

Aim of study	AI methods	Results	REF
Verify the advantages of two-dimensional correlation spectral images (2D-COS) combined with deep learning in identifying herbs	Deep learning, residual convolutional neural network	DL model based on digital image processing is more suitable for identification of different habitats and parts of Panax notoginseng	Liu et al. (2022c)
Predict Quality Markers of Atractylodis Rhizoma [Asteraceae; Atractylodis macrocephalae Rhizoma]	Network pharmacology	Identified four active constituents which can be used as Q-markers of Atractylodis Rhizoma	Zhao et al. (2023)
Explore effective mechanism and quality control of Tongsaimai tablet (TSMT) for anti-atherosclerosis benefit	Network pharmacology	Screened out the potential anti-AS mechanisms and chemical quality markers	Cheng et al. (2022b)
Developed a new strategy of multi-dimensional “radar chart” mode to stablish the holistic quality control of Qiliqiangxin Capsule (QLQX)	Network pharmacology	Discovered the Q-markers of QLQX	He et al. (2021)
Develop a processing-associated quality marker (Q-marker) discovery strategy	Systems pharmacology, *in vivo* high-throughput screening model	Developed a processing-associated Q-marker discovery strategy for carbonized TCM	Gao et al. (2022)
Establish the quality maker evaluation system	Molecular docking	Selected 7 compounds as quality markers of Mume Fructus that could be used for the process quality control	Liu et al. (2022d)
Identify more reasonable markers for quality control of TCM formulas	Network pharmacology analysis, visualization, molecular docking	Successfully established a novel strategy combining intestinal absorption with network pharmacology analysis	Duan et al. (2020)

## 5 Application of AI in TCM industry technology

### 5.1 Auxiliary diagnosis of TCM

The diagnostic methods of TCM doctors include observing, smelling, inquiring, and palpation, all of which rely on the subjective judge and personal experience to evaluate various symptoms. However, the emergence of AI technology provides a new way to strengthen the diagnostic methods of TCM. Image intelligent recognition, computer vision and natural language processing technology provide technical support for the development of AI in TCM diagnosis (Gichoya et al., 2022; Oner et al., 2022). Through big data learning, AI can achieve diagnosis and treatment results that are highly matched with clinical experts. By using human information collection equipment, AI can simulate the diagnosis process of TCM and help doctors identify any missing parts (Zhang et al., 2021a; Xu et al., 2021). In the field of gastric cancer diagnosis, Li et al. developed three AI deep learning diagnosis models based on tongue images, which made great contributions to gastric cancer screening and diagnosis (Yuan et al., 2023). Another research by Lu et al. proposed to combine modern ophthalmology diagnosis technology with AI analysis technology to establish an algorithm model that can identify the relationship between eye color, shape and disease, and finally transform these findings into clinical diagnosis (Lu et al., 2022). In addition, many scholars explored the potential application of AI in TCM pulse diagnosis, and brought new ideas for standardization and digitalization of TCM pulse diagnosis (Asadipour et al., 2020; Yeuk-Lan Alice et al., 2021; Chen et al., 2022; Chowdhury et al., 2023). These studies provide strong support for the further development and application of AI in TCM diagnosis. However, AI may not provide accurate diagnosis under complex cases and special circumstances, which requires the subjective judgment of experienced professionals.

It is worth noting that the 11th Revision of the International Classification of Diseases (ICD-11) covered the chapter of traditional medicine originated from TCM for the first time in 2019, including 150 traditional medical diseases and 196 syndromes. This not only provides a general standard for TCM diagnosis, but also further illustrates the ability and position of TCM services in human health services, which is conducive to promoting the integration and development of TCM and medical and health systems around the world.

### 5.2 TCM education

For thousands of years, there has been a problem of slow speed of inheritance in cultivation of TCM talents. The knowledge system of TCM is extremely complicated, which requires the guidance of experienced experts and the accumulation of clinical wisdom (Li and Graham, 2020). However, the traditional mode of TCM apprenticeship education takes a long time and educates a limited number of talents, making it difficult to meet the growing clinical needs. Moreover, different diagnostic methods and philosophies among various TCM schools and experienced practitioners have brought obstacles to standardization and learning. In recent years, the remarkable progress of AI technology has made the application of AI in TCM education more mature. With the assistance of AI and big data technology, personalized diagnosis and treatment experience of well-known doctors and literature can be transformed into standardized protocols, enriching educational practice and enhancing the clinical acuity of practitioners (Dou et al., 2021; Xu et al., 2023). Intelligent inheritance system of respected doctors’ experience and TCM clinical decision support systems driven by AI have been widely verified (Wenxiang et al., 2022). AI technology has reached a level of proficiency similar to that of TCM clinical experts, and it is helpful to cultivate talented students, thus improving the efficiency of TCM inheritance and promoting its development. Related researches are shown in Table 4. Although AI cannot completely replace clinical practice and guidance in the real world, its combination with TCM education has opened up a new possibility to solve the challenges faced by the cultivation and inheritance of TCM talents.

**TABLE 4:** Application of AI in TCM industry technology.

Aim of study	AI methods	Results	REF
Study the value of tongue picture and tongue coating microflora in the diagnosis of gastric cancer	Deep learning	Developed three artificial intelligence deep learning diagnosis models based on tongue images	Yuan et al. (2023)
Propose an artificial intelligence (AI) system for diagnosis of congenital heart diseases	Computed tomography images, deep learning	Proved the potential of our model to be integrated into current clinic practice to improve the diagnosis of CHD globally	Xu et al. (2023b)
Introduce an automated tool -computer-assisted cardiac cavity tracking (CACCT) to identify complicated cardiac malformations in mouse hearts	Deep learning	CACCT can identify complicated cardiac malformations in mouse hearts automatically	Chu et al. (2020)
report a clinically applicable system to detect gastric cancer	Deep convolutional neural network	The system could aid pathologists in improving diagnostic accuracy	Song et al. (2020)
Propose a graph based multichannel feature fusion (GBMFF) method for wrist pulse diagnosis	Graph convolutional networks	Demonstrated the proposed AI-based method can obtain great performances	Zhang et al. (2021b)
Assist diagnosis of tongue images and realize intelligent tongue diagnosis	U-Net with Global Convolution Network Module	Proposed an improved U-Net network which has a better segmentation effect and higher segmentation accuracy for fissured tongue image dataset	Li et al. (2021)
Construct tongue coating recognition model to assist syndrome diagnosis	Convolutional neural network technique, greasy tongue coating recognition networks (GreasyCoatNet)	Derived a disease-specific deep learning network by finetuning the generic GreasyCoatNet	Wang et al. (2022e)
Construction of Chinese herbal prescriptions from tongue images	Deep learning, CNNs	Verified the feasibility of the proposed method for the automatic construction of herbal prescriptions from tongue images	Hu et al. (2021)
Developed an SPL analysis system for wide-field images	Deep convolutional neural networks (DCNNs)	The method can achieve dermatologist-level detection of suspicious pigmented skin lesions	Soenksen et al. (2021)
Develop an artificial intelligence network to simulate the clinical decision-making of radiotherapy	3D convolutional neural network	Proved the feasibility of artificial intelligence in predicting the dose prescription of CDM radiotherapy	Cao et al. (2023)
Opens an avenue for mental health and evaluates the impact of therapeutic interventions to enhance a holistic state of health	Decision Tree Algorithms, machine learning	Offered a unique approach to characterizing health issues related to psychosomatic health	Morande (2022)
Enhance the explainability of AI applications in healthcare for hospital recommendation	Deep learning, decision trees	Improved the explainability of AI applications in healthcare	Wang et al. (2023c)
Summarize the application of AI in healthcare sector	Machine learning	Although AI holds significant potential for improving patient care, it also presents risks and challenges	Polevikov (2023)

### 5.3 TCM health management

The growing popularity of sub-health conditions has led to a significant change in people’s health concepts. Individuals are no longer only concerned with strengthening disease diagnosis and treatment facilities, but more focus on health management and the improvement of the overall quality of life (Hong et al., 2023). The core concept of TCM is to prevent diseases before they occur. It has obvious advantages in preventing diseases through targeted intervention, which is emphasized by its holistic view and dialectical approach to solving health problems (Pan et al., 2020). This preventive method is highly compatible with modern preventive medicine, which include two key aspects, nip in the bud and attach great importance to early diagnosis and treatment when diseases appear, with the goal of controlling and managing diseases in time. TCM is widely recognized in China and many other countries, and plays a crucial role in disease prevention and healthcare services (Meng et al., 2020).

TCM health management involves health information collection, record establishment, health status assessment and risk prediction. Through personal TCM data collection tools, AI can continuously track and collect personal health information (Kechagioglou, 2023). Based on data mining technology, machine learning and artificial neural network, an intelligent TCM health management system can be established for comprehensive health data analysis (Seibert et al., 2021; Wu et al., 2021). The system can use computer technology to monitor and evaluate personal health in real time to identify abnormal situations and take timely intervention measures (von Gerich et al., 2022; Koski and Murphy, 2021). According to the different physical conditions of individuals, targeted health prescriptions are formulated to assist in accurate medical care (Johnson et al., 2021; Cao et al., 2023). With the rapid development of AI in the field of TCM, researchers are increasingly turning their attention to the health management platform centered on TCM, mainly focusing on diseases prevention (Esmaeilzadeh, 2020). It is worth noting that inaccurate or missing data may affect the accuracy and reliability of AI system. Effective cooperation and synergy between trained TCM professionals and AI can significantly enhance disease prevention and health management.

## 6 Conclusion

The integration of AI into the modernization of TCM represents a promising Frontier that has shown great potential across all aspects of TCM industry. The powerful computing and learning ability of AI technology can significantly accelerate the efficiency of new drug discovery. Using data mining technology to digitize ancient TCM books is helpful to analyze the compatibility and application law of TCM compounds and predict the core drug pairs. In addition, AI technology helps to improve quality standardization of TCM, such as unified resource management and online supervision of TCM decoction pieces through AI cloud platform. Moreover, the big data platform driven by AI collects different data of patients to realize precise medical care and overall patient service, covering diagnosis, treatment and health management. This comprehensive analysis positions AI as the driving force of TCM modernization, which provides an exciting opportunity for the modernization of TCM.

However, the application of AI in TCM industry also faces many problems and shortcomings. The gap in the research of TCM prescription pharmacology quality problems that plague the database of TCM weaken the accuracy and reliability of AI algorithms. In order to overcome these obstacles, it is necessary to strengthen pharmacological research and establish a standardized high-quality database of TCM. The seamless cooperation between AI system and professional practitioners of TCM is very important for the successful application of AI in TCM industry. In addition, the risk of reduced communication between doctors and patients caused by AI intervention makes the design of human-computer interaction and the improvement of user experience an important consideration in the application of AI technology in TCM.

This paper comprehensively summarizes the great progress made by AI technology in various stages of the development of TCM and bridges the gap between traditional wisdom and modern technology, which provides guidance for further exploring the new direction and strategy of AI in the research and application of TCM industry. Although AI technology has brought new opportunities and breakthroughs for the development of TCM, we must be aware of the limitations and challenges of AI technology in the modern application of TCM. Therefore, medical and healthcare scholars should give priority to the research and development of AI technology suitable for TCM industry to promote the modernization of TCM and provide better medical and healthcare services for the general public.
